# GPXplore: an intelligent computational framework for precise gene promoter extraction

**DOI:** 10.3389/fbinf.2026.1740722

**Published:** 2026-02-03

**Authors:** Shruti Godara, Samarth Godara, Shbana Begam, Anil Kumar Singh

**Affiliations:** 1 ICFRE-Forest Research Institute, Dehradun, Uttarakhand, India; 2 ICAR-Indian Agricultural Statistics Research Institute, New Delhi, India; 3 ICAR-National Institute for Plant Biotechnology, New Delhi, India

**Keywords:** downstream, gene regulator, genomics, promoters, upstream

## Abstract

Efficient and precise extraction of gene promoter regions is vital for understanding gene regulation, with broad implications in gene editing, functional genomics, and disease research. However, existing tools often fall short in scalability, usability and performance. To address these limitations, we present “GPXplore,” a computational tool designed for the precise and user-friendly extraction of gene promoters from genomic data. It leverages vectorized data processing techniques to significantly reduce data processing time, enhancing speed and efficiency in large-scale promoter extraction tasks. GPXplore retrieves upstream and downstream sequences relative to gene loci and supports customizable parameters, enabling users to define region lengths based on specific research needs. The tool is implemented in Python, features both a command-line and graphical user interface, and is compatible with Windows and Ubuntu platforms. GPXplore was rigorously validated using eight diverse genomic datasets, demonstrating high accuracy and reliability. By combining automation, flexibility, and accessibility, GPXplore provides a robust solution for researchers across varying levels of computational expertise, facilitating high-throughput promoter analysis in modern genomics.

## 1 Introduction

In the exponential landscape of omics, understanding genes, the genomic elements that encode proteins essential for cellular function and development is foundational. Elucidating gene function helps uncover the mechanisms that drive normal biological processes as well as disease pathogenesis (Romero et al., 2012). However, gene expression is not uniform but it is precisely controlled through gene regulation, a set of mechanisms that determine when, where, and how much a gene is expressed, ensuring that proteins are produced correctly and at appropriate times.

**FIGURE 1:**
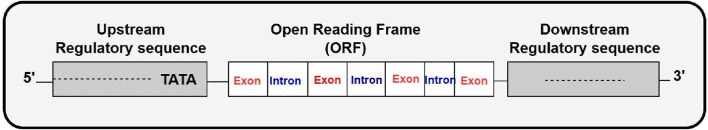
Graphical illustration of the structure of a gene, including the promoter region.

In this scenario, the gene promoter is the central component of the gene regulation process. A gene promoter is a specific region of nucleotide sequences that initiates transcription, the process by which an RNA copy is made from a DNA template (Preker et al., 2011). Typically located upstream of the coding region in the 5′direction (just before the start of the coding sequence of a gene), promoters contain conserved sequence motifs such as the TATA box, CAAT box, and GC rich regions which help guide the transcriptional machinery (Bergman et al., 2022). In some cases, regulatory elements may also exist downstream of the transcription start site. Figure 1 illustrates the general structure of a gene, highlighting the location and key elements of the promoter region to provide foundational context for the study.

Understanding promoter structure and sequence is vital for deciphering gene regulation and is often the first step in designing gene expression studies or engineering genetic mutations (Andersson and Sandelin, 2020). Traditionally, this involves *in silico* validation followed by PCR-based confirmation.

Despite its importance, identifying gene promoters across a genome remains a complex task. Genomic datasets typically contain millions of base pairs and thousands of genes, making manual promoter identification impractical. This process demands extensive effort and precision, as it involves identifying specific regulatory sequences within the genome that initiate gene transcription. The manual approach is not only labour-intensive but also prone to errors, rendering it inefficient for managing the large datasets typically encountered in genomic research.It demands extensive effort, is error-prone, and lacks scalability. In response, several computational methods have been developed to streamline promoter extraction.

Several existing tools and databases provide access to promoter-related information, but they each come with significant limitations. For instance, the UCSC Genome Browser (Karolchik et al., 2011) and NCBI Entrez (Brown et al., 2015) offer access to annotated genomic regions; however, they are limited to genes already included in their repositories. This means they cannot retrieve promoter sequences for novel or newly identified genes that have not yet been annotated in their respective databases. Furthermore, both platforms operate on a single-gene query basis, which makes them inefficient for large-scale or batch promoter extraction tasks. Databases such as PlantPAN (Chow et al., 2024) and PlantCARE (Lescot et al., 2002) are valuable resources for motif-level promoter analysis, focusing on the identification of regulatory elements like transcription factor binding sites. However, they do not provide scalable functionality for batch promoter sequence extraction across multiple genes, limiting their use in high-throughput studies.

The GenomicFeatures package in R (Lawrence et al., 2013) offers a more flexible and programmatic approach for promoter extraction by allowing users to manipulate genomic annotations and extract upstream regions. While powerful, this method assumes a working knowledge of the R programming language, which presents a steep learning curve and limits accessibility for researchers without coding experience. These limitations collectively highlight the need for an efficient, scalable, and user-friendly tool like GPXplore that bridges these gaps in functionality and usability.

These limitations underscore the need for an intuitive, fast, efficient, and scalable tool for promoter extraction that enables bulk promoter extraction without requiring programming expertise. To address this, the presented study introduces GPXplore, a GUI-based computational tool that enables efficient extraction of gene promoters for multiple genes simultaneously. GPXplore efficiently extracts user-defined upstream and downstream promoter regions using high-performance techniques, including parallel processing. Its structured tabular output ensures easy interpretation and supports advanced regulatory analysis.

The software is built on a robust algorithm that ensures high accuracy in promoter identification. It provides outputs in a structured tabular format, facilitating interpretation and integration into downstream analysis workflows. Designed with accessibility in mind, GPXplore requires no installation, is compatible with both Windows and Ubuntu, and is available as open-source software for full transparency and customisation.

To validate its efficacy, GPXplore was rigorously tested on eight distinct datasets, demonstrating its high accuracy and reliability in promoter extraction. This extensive testing confirms the software’s robustness and makes it a reliable choice for genomic researchers requiring precise and scalable gene promoter analysis tools. By addressing limitations found in existing tools, such as lack of scalability, speed, ease of use, and accuracy, GPXplore offers an advanced, effective solution for gene promoter extraction, making it a valuable resource for both novice and experienced genomic researchers alike.

## 2 Methodology

**FIGURE 2:**
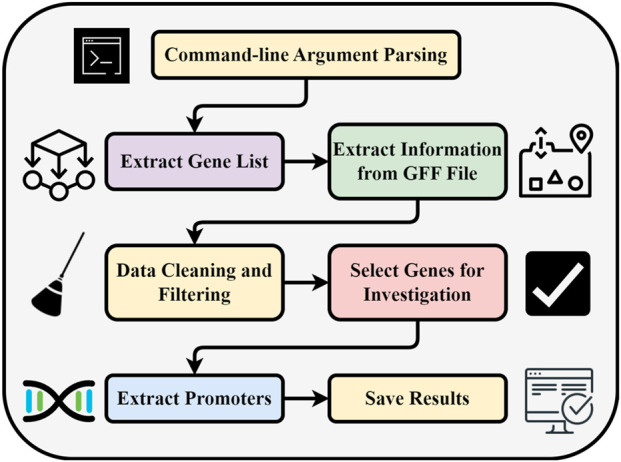
Methodological workflow of the GPXplore.

GPXplore is a computational tool designed to automate the extraction of gene promoter sequences from genomic data. It integrates a command-line script with a graphical user interface (GUI), offering ease of use and high performance through Python-based optimizations. The methodological pipeline is visualized in Figure 2.

### 2.1 Input requirements

GPXplore requires three primary input files for its operation: a genome FASTA file containing the reference genome sequence, a gene FASTA file that includes the identifiers of the genes of interest, and a GFF file, which provides the necessary genomic annotations such as feature locations and gene structures. In addition to these, users can specify optional parameters defining the lengths of upstream and downstream regions to be extracted, with a default setting of 2000 base pairs for each. These inputs can be provided either through the command-line interface or the GUI, offering flexibility based on the user’s preference and level of technical expertise.

### 2.2 Promoter extraction workflow

The GPXplore tool automates promoter sequence extraction through a structured multi-step pipeline (Figure 2). It begins by using the argparse module in Python to parse essential command-line inputs, including genome and gene FASTA files, a GFF annotation file, and output preferences. Optional parameters allow users to set upstream and downstream sequence lengths. The tool then extracts gene IDs from the gene FASTA headers and processes the GFF file to collect genomic features such as chromosome, position, and feature type. It performs data cleaning by matching gene IDs across files and retaining relevant entries labeled as gene or mRNA. After selecting a single representative record per gene, GPXplore retrieves promoter regions from the genome based on specified coordinates. The final output, containing promoter sequences and metadata, is saved in a structured CSV file for easy downstream analysis.

### 2.3 Algorithm process optimization

Based on the described workflow, GPXplore incorporates a novel algorithm that emphasizes its technical structure, providing developers and advanced users with a clear understanding of the precise functional implementation of promoter extraction. To ensure scalability and high-speed execution, GPXplore leverages vectorized data processing using the *“apply”* function from the Pandas library. This function helps to operates on entire data columns at once, reducing iteration time, minimizes overhead from Python loops by using optimized C/Cython backend, enables faster computation by supporting multi-threaded or multi-process execution and optimizes memory and cache access patterns, making it suitable for large datasets. These features significantly accelerate the promoter extraction process compared to conventional looping methods.

### 2.4 Software architecture and GUI integration

GPXplore adopts a modular architecture, separating logic into reusable components such as Input parsing, Gene filtering, Promoter extraction and Output generation. This modularity facilitates easy maintenance and future upgrades. The core Python script was extended with a GUI developed using the Tkinter framework (Amos, 2020), enabling intuitive use for researchers without coding experience. The GUI allows users to load files, set parameters, and view logs, making the tool accessible across expertise levels.

### 2.5 Promoter extraction algorithm

In this study, a novel algorithm was developed to automate the extraction of gene promoter sequences from genomic data. The algorithm serves as the computational core of the GPXplore tool and is presented through detailed pseudocode in (Supplementary Sheet). This algorithm systematically outlines the step-by-step process for promoter extraction and optimization of tool functionality. It is implemented using several custom-built functions, each dedicated to a specific stage of the pipeline. Functions such as extract_gene_ids (), extract_gff_info (), preprocess_gids (), filter_features (), and extract_streams () were designed from scratch to ensure efficiency and adaptability. Their modular design enhances flexibility, accuracy, and independence from third-party dependencies. Additionally, this modular architecture improves code maintainability, allowing easy integration with other bioinformatics frameworks in the future.

### 2.6 Dual-interface application

**FIGURE 3:**
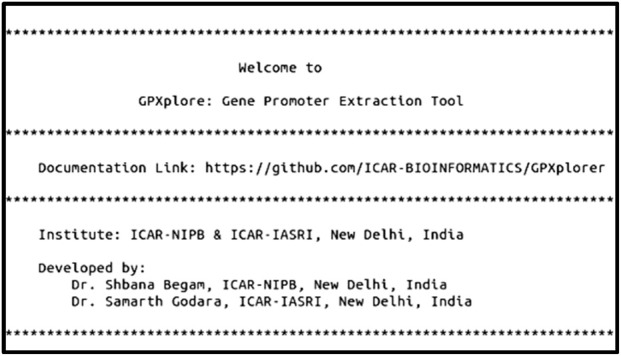
Commands to run GPXplore using the command-line interface.

**FIGURE 4:**
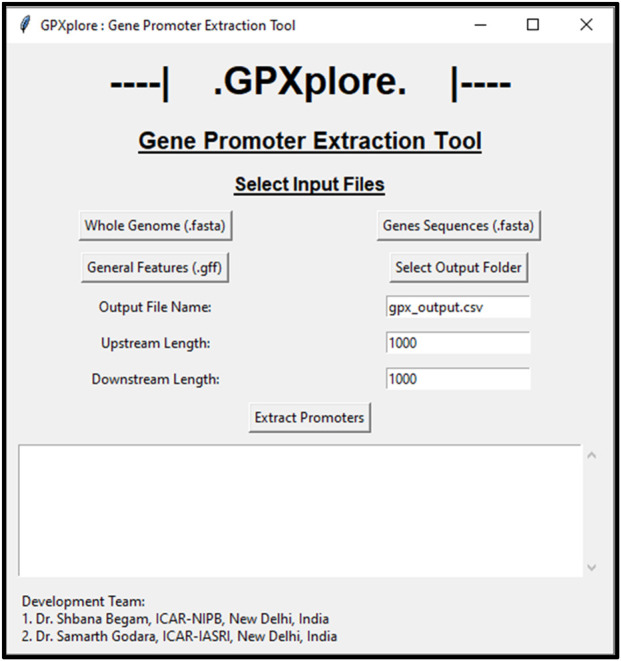
Home screen of GPXplore’s graphical user interface.

Using the designed algorithm as its computational backbone, the GPXplore tool was developed in two user-accessible formats: a command-line interface and a graphical user interface. The command-line version enables advanced users to execute batch promoter extraction efficiently with customizable parameters, offering flexibility and automation for large-scale genomic workflows (Figure 3). Simultaneously, the GUI was built using the Tkinter framework to ensure ease of use for researchers with limited programming experience (Figure 4). The GUI provides an intuitive environment to upload input files, define promoter lengths, and retrieve results with minimal manual effort. This dual-interface architecture, powered by the same underlying algorithm, ensures that users who are comfortable with coding can benefit from the streamlined, script-based functionality of the command line, while those with less programming experience can easily navigate the tool using the intuitive GUI. The flexibility of these options enhances the accessibility and usability of GPXplore, making it a valuable tool for a broad spectrum of researchers. Screenshots illustrating both interfaces are provided.

The graphical user interface of GPXplore has been thoughtfully designed to be compatible with both Windows and Linux operating systems, ensuring accessibility for a wide range of users across different platforms. This cross-platform capability enhances the tool’s usability and makes it easier for researchers from diverse technical backgrounds to engage with the software, facilitating broader adoption and integration into various genomic studies.

The final output irrespective of interface is a structured CSV file containing Gene ID, Chromosome number, Start and end positions and Extracted upstream and downstream sequences. This output can be directly used for downstream applications such as primer design for PCR amplification.

## 3 Experiments and results

### 3.1 Experimental setup

To evaluate the performance and versatility of GPXplore, a comprehensive experimental setup was established using datasets from eight diverse crop species. These datasets were retrieved from publicly accessible and credible genomic databases, such as NCBI (Federhen, 2012) and Phytozome (Goodstein et al., 2012), ensuring the authenticity and completeness of the genomic information used for testing. Each dataset included three essential input files required by GPXplore: genome FASTA file containing the complete reference genome sequence, gene FASTA file listing the gene identifiers of interest, and GFF file providing annotations of genomic features, including gene loci and structure. All the experiments in the present study were executed on a computational unit with Ubuntu 21.10 operating system on Intel Xeon(R) Gold 6226R CPU @ 2.90GHz × 64 microprocessor, NVIDIA-352.63 and 128 GB of primary memory with 8 TB of secondary memory. The GPXplore software is implemented in Python (version 3.10), utilizing the argparse, pandas and Biopython libraries.

The selected crop species represent a wide taxonomic range, allowing for the validation of the tool across genomes of varying sizes and complexities. Details of the experimental datasets, including genome sizes and total number of genes, are summarized in Table 1. Table information explains variation in genome sizes and total gene numbers. This comparative visualization provides insight into the range of genomic complexities handled by GPXplore during testing, demonstrating its scalability and applicability across diverse plant genomes.

**TABLE 1:** Details of experimental data.

S.No.	Name of crop	Genome size	Total genes
1	*Berberis vulgaris*	540.9 MB	24,255
2	*Brassica oleracea*	391.4 MB	35,400
3	*Cicer anatolicum*	537.8 MB	28,269
4	*Glycine soja*	1.0 GB	62,102
5	*Oryza sativa*	382.0 MB	52,424
6	*Phaseolus acutifolius*	521.2 MB	50,635
7	*Spinacia oleracea*	928.8 MB	34,875
8	*Sorghum bicolor*	688.6 MB	49,348

In addition to these inputs, the promoter extraction was guided by user-defined parameters specifying the lengths of upstream and downstream sequences to be extracted. By default, GPXplore uses 2,000 base pairs in both directions from the transcription start site, although this value can be customized depending on specific research requirements.

### 3.2 Performance evaluation

Unlike existing tools that typically allow the extraction of one promoter at a time and rely solely on their own pre-integrated reference genome databases, GPXplore offers the flexibility to work with user-provided reference genomes and gene sets. This is particularly important in research scenarios where scientists are working with newly sequenced or custom-assembled genomes that are not available in public repositories. GPXplore supports bulk promoter extraction by allowing users to input their own genome FASTA, gene FASTA, and GFF annotation files, enabling high-throughput analysis across thousands of genes in a single run. Therefore, the performance evaluation of GPXplore was conducted using results generated from user-supplied reference genomes and annotations, rather than relying on pre-existing tool databases. This approach not only reflects real-world research scenarios more accurately but also demonstrates the tool’s robustness and adaptability across diverse, user-defined genomic contexts.

**FIGURE 5:**
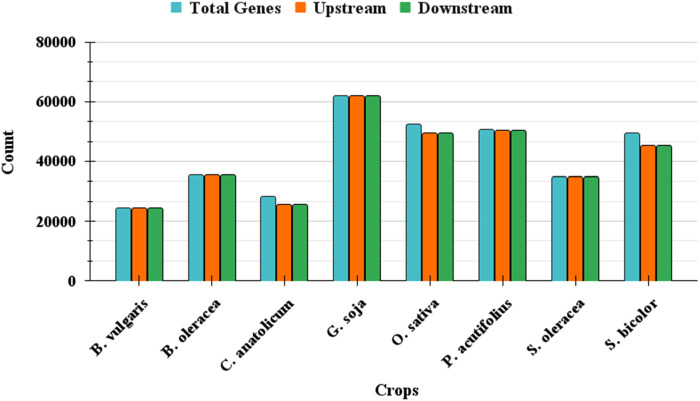
Number of extracted promoters using GPXplore.

The performance of GPXplore was assessed using datasets mentioned in the experimental set up section. The tool was evaluated for its efficiency and accuracy in extracting upstream and downstream promoter sequences based on specified gene inputs and genomic annotations. For each species, the total number of genes was provided as input, and the tool was tasked with extracting the respective promoter sequences. As summarized in Table 2, GPXplore successfully extracted a high number of upstream and downstream sequences for most datasets. Figure 5 visually depicts the number of promoters successfully extracted for each crop where the bar for promoters and their respective genes are almost similar.

**TABLE 2:** Number of upstream and downstream sequences using GPXplore.

S.No.	Name of crop	Total no. of genes	No of upstream extracted	No of downstream extracted
1	*Berberis vulgaris*	24,255	24,252	24,255
2	*Brassica oleracea*	35,400	35,400	35,400
3	*Cicer anatolicum*	28,269	25,552	25,583
4	*Glycine soja*	61,806	61,804	61,806
5	*Oryza sativa*	52,424	49,416	49,416
6	*Phaseolus acutifolius*	50,635	50,220	50,218
7	*Spinacia oleracea*	34,876	34,862	34,876
8	*Sorghum bicolor*	49,348	45,203	45,203

During testing, GPXplore successfully extracted promoter sequences for the majority of genes in each crop. However, minor discrepancies in promoter extraction occurred when genes were located too close to chromosome ends or within regions lacking sufficient flanking sequences to meet the default 2000 bp promoter length requirement. These limitations are inherent to the genomic data structure and do not reflect tool inefficiency.

### 3.3 Statistical analysis

To assess the accuracy of GPXplore, the error rate was calculated for each dataset using the following formula (Equation 1):

$$Error\;Rate=\frac{\left|Total\;No\;of\;Genes-No\;of\;Promoters\;\left(up\;or\;down\right)\;Extracted\right|}{Total\;No\;of\;Genes}\times 100$$ (1)

This statistical measure indicates the percentage of genes for which promoter sequences could not be extracted, often due to genomic constraints such as gene location near chromosomal boundaries.

**FIGURE 6:**
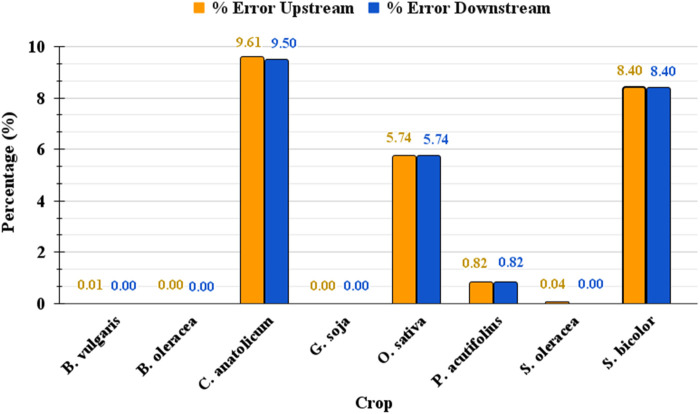
Comparative error rates in promoter sequence extraction by GPXplore.

Across all tested datasets, the average error rate for upstream promoter extraction was 3.07%, with a minimum of 0% and a maximum of 9.61%. For downstream promoters, the average error rate was 3.05%, ranging from 0% to 9.50%. This evaluation, visualized in Figure 6, underscores GPXplore’s capability to accurately and efficiently process large-scale genomic datasets with minimal error. The high extraction accuracy and automated functionality of the tool make it highly suitable for high-throughput promoter analysis across diverse plant genomes.

## 4 Discussion

The development of GPXplore marks a substantial contribution to the computational genomics landscape, particularly in gene promoter analysis. Traditional approaches, such as genome browsers (e.g., UCSC Genome Browser or manual extraction using BEDtools (Quinlan and Hall, 2010), are often labor-intensive and constrained when applied to large or fragmented genomes. Several tools like PlantPAN, PlantCARE, and PLACE (Higo et al., 1998) provide motif-level analysis but lack scalable functionality for batch promoter extraction. GPXplore addresses this gap by offering a streamlined, high-throughput pipeline capable of extracting both upstream and downstream promoter sequences across diverse plant genomes.

In our comparative evaluation across eight plant species of varying genome sizes, GPXplore consistently demonstrated high accuracy (mean error rates under 5%). The tool’s failure to extract promoters in a minority of cases was primarily due to chromosomal boundary conditions—an issue also acknowledged in genome-scale tools like BEDtools and HOMER (Mestre et al., 2013), which similarly face challenges in edge cases near contig boundaries or scaffolds.

Unlike existing tools limited to specific genomes or requiring extensive formatting, GPXplore is agnostic to organisms and format-flexible, making it suitable for non-model species with fragmented assemblies. For example, its successful application to *Glycine soja*, which includes over 60,000 genes, illustrates its scalability, complementing recent efforts in promoter analysis in large-genome crops like wheat (Ramírez-Gonzálezet al., 2018). In addition to its broad applicability, GPXplore is equipped with a novel, modular algorithm designed to optimize promoter extraction with high precision and efficiency. Leveraging parallel processing and optimized data structures, GPXplore delivers faster runtime performance even with complex genome assemblies. Furthermore, the tool’s structured, tabular output supports compatibility with downstream bioinformatics workflows, facilitating comparative and functional genomics studies.

The flexibility to customize upstream/downstream lengths (default: 2,000 bp) aligns GPXplore with variable regulatory distances observed in literature (Yamamoto et al., 2010), thereby enhancing its applicability in transcription factor binding site (TFBS) prediction and motif scanning. While current versions focus solely on sequence extraction, future iterations will integrate motif discovery and statistical analysis modules to support end-to-end regulatory genomics workflows, similar to integrated platforms like MEME Suite (Bailey et al., 2009).

Moreover, a direct, quantitative performance comparison between GPXplore and existing tools is not feasible for several reasons. Existing resources such as PlantPAN, PlantCARE, and PLACE are primarily motif and TFBS analysis platforms or motif databases, not dedicated high-throughput promoter extractors, so their core objectives and performance metrics differ fundamentally from GPXplore’s extraction-focused design. Many of these tools operate as web servers with opaque back-end implementations, making it impossible to benchmark runtime, memory usage, or scalability under controlled, reproducible conditions comparable to a standalone, script-based tool like GPXplore.

Furthermore, BEDtools and similar command-line utilities are general-purpose genomic interval manipulators rather than specialized promoter-extraction frameworks, and they require substantial manual preprocessing and scripting, which introduces user-dependent variability that cannot be standardized for fair comparison with GPXplore’s automated pipeline. Some widely used platforms, such as MEME Suite, assume that promoter or peak sequences have already been extracted and focus solely on motif discovery and statistical analysis, placing them downstream of GPXplore in the analysis pipeline rather than in the same functional category for head-to-head performance evaluation.

The potential applications of GPXplore extend across diverse realms of biological inquiry, owing to its versatility and efficacy in gene promoter extraction. In systems biology, extracted promoters can be used to reconstruct gene regulatory networks. Furthermore, GPXplore finds utility in comparative genomics and evolutionary studies, where the identification and comparison of gene promoters across species offer valuable insights into evolutionary conservation and divergence. Researchers can discern conserved regulatory elements and evolutionary signatures by systematically extracting upstream and downstream sequences, shedding light on the evolutionary dynamics shaping genome architecture and function. Furthermore, the tool’s GUI and CLI dual-interface ensures accessibility for both novice users and bioinformaticians.

## 5 Conclusion

GPXplore is a powerful and versatile tool designed for efficient and accurate extraction of gene promoter sequences from large genomic datasets. By offering both a command-line interface (CLI) and a graphical user interface (GUI) with cross-platform capability, it caters to a wide range of users, from bioinformatics professionals to researchers with limited programming skills. The tool has been rigorously tested across multiple crop genomes, demonstrating high accuracy and scalability. Its ability to handle large datasets, extract upstream and downstream sequences with precision, and overcome the limitations of existing promoter extraction tools makes GPXplore an indispensable resource for genomic research. Moreover, GPXplore’s code is available online, allowing researchers to build upon and extend the tool for further exploration in gene promoter extraction. The authors plan to incorporate statistical analysis functionality in future versions to offer a more comprehensive solution for promoters’ processing. Overall, as a user-friendly and flexible solution, GPXplore not only streamlines the promoter extraction process but also paves the way for deeper insights into gene regulation and functional genomics.

## Data Availability

Publicly available datasets were analyzed in this study. This data can be found here: https://github.com/ICAR-TECH/GPXplore.
