# Supplementary material for: GPXplore: an intelligent computational framework for precise gene promoter extraction
Source: Front Bioinform. 2026 Feb 3;6:1740722. doi: 10.3389/fbinf.2026.1740722 (PMC12909496; doi:10.3389/fbinf.2026.1740722)
Supplement: Supplementary file 1 [file DataSheet1.pdf]

<u>Supplementary Sheet</u>

GPXplore: An Intelligent Computational Framework for Precise Gene Promoter Extraction

Promoter Extraction Algorithm

In this study, a novel algorithm was designed and developed to automate the extraction of gene promoter sequences from genomic data. The algorithm outlines a systematic procedure, presented through detailed pseudocode, that forms the core computational engine of the GPXplore tool. Algorithm 1 shows the pseudocode for the GPXplore tool.

Algorithm 1: outlines the step-by-step process used in the development of GPXplore, detailing the procedures involved in extracting gene promoter sequences and refining the tool's functionalities for optimal performance.

Algorithm 1 *GPXplore_Gene_Promoter_Extraction*()

```
Input ← Genome FASTA file, Gene FASTA file, GFF file, Output CSV file
    path, Optional parameters (upstream/downstream lengths)
Output ← CSV file with promoter sequences and relevant information
input_parameters ← parse(command_line_arguments)
gene_ids ← extract_gene_ids(gene_fasta)
gff_info ← extract_gff_info(gff_file)
pr_gids ← preprocess_gids(gene_ids)
fltd_feat ← filter_features(gff_info)
for each gene from the pr_gids do
    results ← extract_streams(gene_ID)
end for
save_csv(results, output_file_path)
```

The algorithm is implemented using multiple custom-built functions, specifically designed to handle different stages of the promoter extraction pipeline. Functions such as *extract_gene_ids(), extract_gff_info(), preprocess_gids(), filter_features(),* and *extract_streams()* were developed from scratch to ensure flexibility, performance, and accuracy across diverse genomic datasets. These modular functions enable efficient parsing, cleaning, and processing of complex genomic data and annotations, thereby allowing GPXplore to operate independently of third-party tool dependencies. This function-level modularity not only improves code maintainability and readability but also allows for future upgrades or integration with other bioinformatics pipelines.
